# Kinkéliba (*Combretum micranthum*) Leaf Extract Alleviates Skin Inflammation: In Vitro and In Vivo Study

**DOI:** 10.3390/molecules28041791

**Published:** 2023-02-14

**Authors:** Shuting Hu, James E. Simon, Mingfu Wang, Yizhen Wu, Yumin Huang, Qingli Wu

**Affiliations:** 1 Research & Innovation Center, Acaderma Inc., San Francisco, CA 94111, USA; 2 Rutgers Core Facility for Natural Products & Bioanalysis and The New Use Agriculture and Natural Plant Products Program, Department of Plant Biology, School of Environmental and Biological Sciences, Rutgers University, 59 Dudley Road, New Brunswick, NJ 08901, USA; 3 School of Biological Science, The University of Hong Kong, Pokfulam, Hongkong, China

**Keywords:** kinkéliba, *Combretum micranthum*, anti-inflammation, anti-oxidant, skin repair

## Abstract

Kinkéliba (*Combretum micranthum*, Seh-Haw in Wolof) is a popular bush tea in West African countries. Although the kinkéliba plant’s leaves have been widely consumed for its nutritional and medicinal properties, its benefits on skin health potential have been practically untouched. In human epidermal primary keratinocytes, vitexin and isovitexin-rich kinkéliba extract treatment significantly (*p* < 0.001) enhanced up to 39.6% of the cell survival rate decreased by UV radiation irritation. The treatment of kinkéliba leaf extracts also reduced the production of UV-induced pro-inflammatory cytokines IL-6 and IL-8 by 57.6% and 42.5%, respectively (*p* < 0.001), which cause skin redness and skin barrier dysfunction, as well as wrinkles and collagen degradation. The anti-inflammation efficacy of kinkéliba leaf extracts might involve significant inhibition on the levels of cellular reactive oxygen species (ROS) (−70.8%, *p* < 0.001) and nitrotyrosine (−56.9%, *p* < 0.05). Further topical applications of kinkéliba leaf extract gel were found to reduce sodium lauryl sulfate (SLS)-induced skin inflammation: at D7, the skin trans-epidermal water loss (TEWL) and skin redness (a* value) were both reduced by 59.81% (*p* < 0.001) and 22.4% (*p* < 0.001), compared with D0. In vitro and in vivo data support a new topical application of the kinkéliba leaf as an effective active ingredient for the treatment of skin inflammation, as well as subsequent barrier dysfunction and inflammaging.

## 1. Introduction

*Combretum micranthum* (also known as kinkéliba) is a non-domesticated shrub species found in the jungle in West Africa [1]. It is a dense shrub or vine, commonly found in cultivated and fallow fields, mainly located in sub-Saharan Africa, with higher yields in Senegal, Mali, and Burkina Faso. In several tropical West African savannah countries, people get the leaves from kinkéliba of wild populations as a popular traditional herbal tea. As a shrub tea, kinkéliba has a pleasant taste, and the soup thereof has a color of light to dark greenish-brown. The shrub tea is also used locally as a traditional panacea useful for promoting diuresis and alleviating digestive problems, such as gastrointestinal problems, colic, and vomiting [2,3].

The kinkéliba leaf is rich in flavonoids, including vitexin, isovitexin, orientin, homoorientin, myricetin-3-O-glucoside, and myricetin-3-O-rutinoside, as well as alkaloids including stachytine, hydroxystachyline, and choline [4,5,6]. Our previous studies also identified a group of novel-skeleton flavan-alkaloids (TPFAs), including kinkeloids A, B, C, and D, as unique components from kinkéliba [7]. These new compounds, or kinkéliba extracts containing TPFAs, were considered as potential anti-diabetic agents in the treatment of metabolic disorders. Its glucose-lowering activities were validated in mice in a dose-dependent manner without significant weight loss and toxicity [8]. Kinkéliba’s clinical efficacy also includes normalizing blood pressure and nephroprotection, in recent studies [9,10]. The anti-oxidant and anti-inflammatory effects were examined in cell-free models as well [11,12,13]. Therefore, the kinkéliba leaf extract has the great potential to promote health benefits on skin.

As the largest organ of humans, the skin has three main layers: the epidermis, the dermis, and the subcutaneous layer [14]. It is an effective barrier that protects humans against mechanical, thermal, and physical injury and hazardous substances. It also prevents loss of moisture and acts as a sensory organ. With increasing age, the skin undergoes changes both in appearance and function, and these are exaggerated by exposure to external factors, such as ultraviolet radiation (UVR) [15]. UV radiation is classified as UVA, UVB, or UVC according to the wavelength. Most UVC can be absorbed by the ozone layer before reaching the earth surface, while both UVA and UVB are able to penetrate the atmosphere and cause cumulative injury to the skin [16]. Early biological responses to UVA radiation, including erythema and redness of the skin, are the most prominent visible sign of inflammation [17]. UV-induced inflammation stimulates ROS generation, elevates pro-inflammatory cytokines formation (TNF-α, IL-6, IL-8), and up-regulates the expression of COX-2 and prostaglandin metabolites, especially PGE2 [18,19,20]. The higher circulating levels of cytokines, chemokines, and acute-phase proteins, as well as the greater expression of genes involved in inflammation, will ultimately cause collateral damage to tissues and organs over time [21]. In the epidermis, the existence of chronic inflammatory factors damage the skin barrier, which induces redness, dryness, or inching [22]. In the dermis, inflammatory factors are the main factors that cause the breakdown of the normal skin architecture, including loss of collagen and hyaluronic acid, as well as other glycosaminoglycans including chondroitin. Collagen and elastic fiber degradation and disorganization in the dermis finally results in decreased skin elasticity wrinkles [23]. This chronic, low-grade systemic inflammation, termed inflammaging, is also responsible for the decreased skin repair ability, as well as the slowing down of the skin metabolism [24]. The signs of inflammaging are similar to skin aging, but more specifically, the skin turns to be more sensitive. Itchiness, dryness, and redness are more likely to happen [25].

Chronic inflammation also produces free radicals, which ultimately create more inflammation. This interrelation between oxidative stress and inflammation leads to a “toxic” feedback system [26]. The imbalance between the production of reactive oxygen species (ROS) and the availability of free radical scavengers increases cellular oxidative stress, which triggers signaling cascades that can lead to the onset and progression of inflammation [27]. In addition to ROS, nitric oxide, nitric dioxide, and reactive nitrogen species (RNS) are predominantly associated with inflammatory processes early in cutaneous immune responses [28]. RNS were also suggested to play a role in the induction of matrix metalloproteinases, leading to the degradation of collagen and elastin [29].

Both inflammation and oxidative stress are interrelated since one could promote the other. Therefore, the application of antioxidants and anti-inflammation compounds have been demonstrated to be an effective strategy to alleviate skin inflammation. Experimental and epidemiologic studies have suggested that polyphenols from natural sources protect the skin from free radicals and pro-inflammatory cytokines [16]. Vitexin, an apigenin flavone glycoside, was unambiguously identified as the key compound from kinkéliba leaves from our previous studies [4,5]. A rapid isolation method of vitexin from the leaves of kinkéliba was also established [4,5]. Using vitexin as the key marker, we prepared a vitexin and isovitexin-rich kinkéliba leaf extract to further study kinkéliba’s benefits on skin health. In this investigation, UVR and SLS (sodium lauryl sulfate) were used to induce skin inflammation in vitro and in vivo, respectively. In the meantime, kinkéliba extracts’ efficacy on free radical scavenging and pro-inflammatory cytokines, as well as the repair of skin barrier dysfunction and redness, were evaluated in vitro and in vivo.

## 2. Results

### 2.1. Definition of UVA Dosage

The adverse effects of UVA to keratinocytes were monitored in a dosage range of 0–7.5 J/cm^2^, according to the previous study [30]. Once UVA irradiation was ≥4.5 J/cm^2^, the cell viability was significantly reduced compared with that of the control cells (Supplemental Material Figure S1). Therefore, 4.5 J/cm^2^ was selected as the optimal irradiation dose, as the cell viability was statistically affected and the survival rate exceeded 85%. This is equivalent to about 12–15 min of exposure to noon summer sunlight in Los Angeles [31], Hong Kong [32], Sydney [33], and Singapore [34].

### 2.2. Kinkéliba Leaf Extract Enhanced Cell Viability after UVA Irradiation

To evaluate the potential UVA-protective effects of kinkéliba leaf extracts, human epidermal primary keratinocytes (within passage 5) were cultured and treated with or without kinkéliba leaf extracts for 24 h before irradiation. The kinkéliba leaf extract was tested at concentrations from 2.5 to 100 μg/mL.

**Figure 1:**
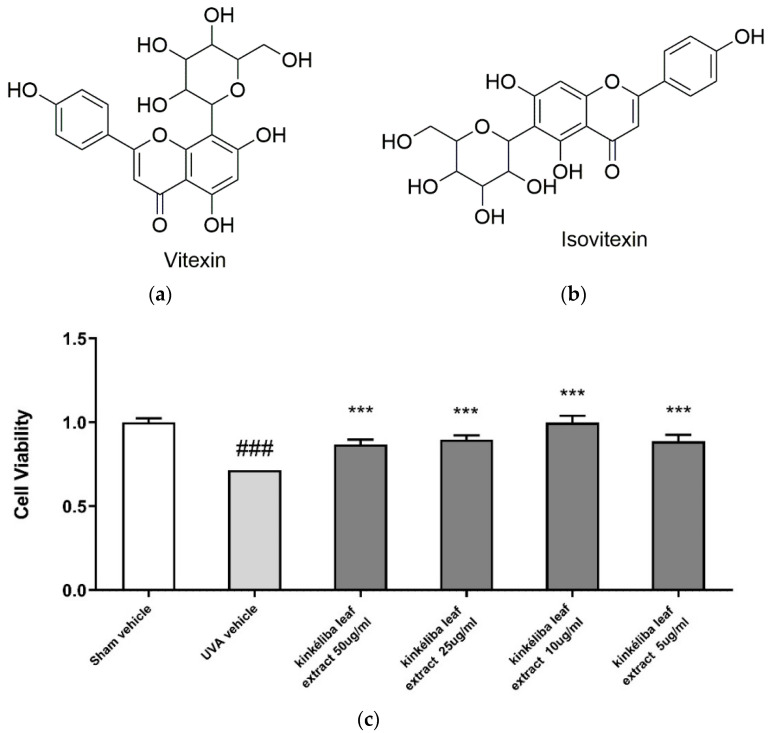
Chemical structure of vitexin (**a**) and isovitexin (**b**); UV protective effect of kinkéliba leaf extract on cell survival rate (**c**). Human primary keratinocytes were treated with kinkéliba leaf extract at different concentrations and cultured for 24 h. Cell viability was determined by the CCK-8 assay. Each value is presented as the mean ± SD from triplicate independent experiments. Significantly different from sham vehicle, ### *p* < 0.001; UVA vehicle, *** *p* < 0.001.

According to HPLC analysis, the concentrations of vitexin and isovitexin were identified as 1.4% and 2.58%, respectively (Supplemental Material Figure S2). The cell viability result showed that the vitexin and isovitexin-rich kinkéliba leaf extract enhanced the cell survival rate significantly, compared with the UVA vehicle, indicating that kinkéliba leaf extracts provided effective protection to human primary keratinocytes (Figure 1c).

### 2.3. Kinkéliba Leaf Extract Decreased UVA-Induced Inflammatory Cytokines Production

**Figure 2:**
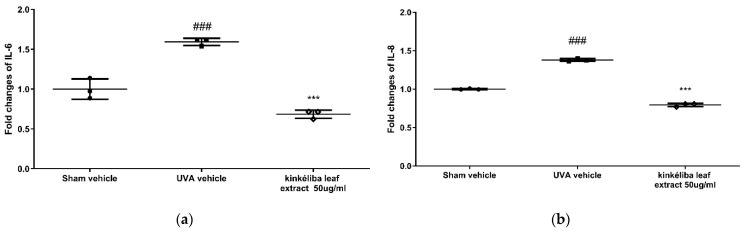
Treatment reduced UVA-induced inflammatory cytokines. Cells were treated with kinkéliba leaf extract and cultured for 24 h after UVA irradiation. The formation of inflammatory cytokines IL-6 (**a**) and IL-8 (**b**) were evaluated by means of ELISA. Each value is presented as the mean ± SD from triplicate independent experiments. Significantly different from sham vehicle, ### *p* < 0.001; UVA vehicle, *** *p* < 0.001.

Inflammatory cytokines indicate the acute inflammatory response induced by UVA. Compared with the UVA vehicle, post-treatment with kinkéliba leaf extracts for 24 h at 50 μg/mL significantly reduced the UVA-induced increase in IL-6 (Figure 2a) and IL-8 (Figure 2b). However, there was no significant decreases on the UVA-induced TNF-α level.

### 2.4. Kinkéliba Leaf Extract Reduced UVA-Induced ROS Production

**Figure 3:**
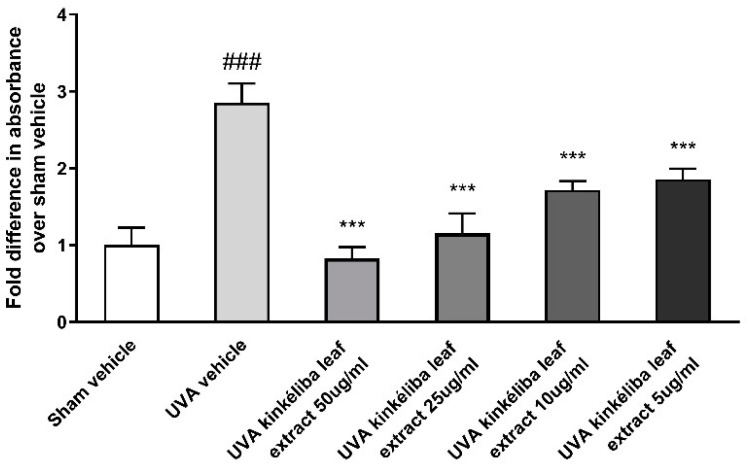
Treatment reduced UVA-induced reactive oxygen species (ROS). Cells were treated with kinkéliba leaf extract and cultured for 24 h before UVA irradiation. The formation of ROS was evaluated by means of DCFA. Each value is presented as the mean ± SD from triplicate independent experiments. Significantly different from sham vehicle, ### *p* < 0.001; UVA vehicle, *** *p* < 0.001.

UV radiation is a major environmental threat to the skin. It leads to increases in photo-oxidative damage by the generation of reactive oxygen species (ROS) [35]. Kinkéliba leaf extracts were used at the concentration of 5 μg/mL and 10 μg/mL, 24 h prior to irradiation with UVA. Compared with the sham vehicle, the UVA-vehicle-treated group significantly increased the production of cellular ROS. Meanwhile, pretreatment with the kinkéliba leaf extract (Figure 3) significantly reduced the UVA-induced increase in ROS, indicating that these agents could protect the cells from ROS damage.

### 2.5. Kinkéliba Leaf Extract Protected against UVA-Induced Increase in Nitrotyrosine

**Figure 4:**
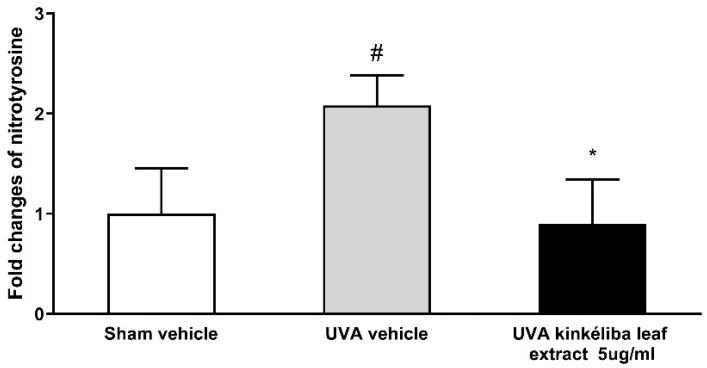
Treatment reduced UVA-induced nitrotyrosine. Cells were treated with kinkéliba leaf extract and cultured for 16 h after UVA irradiation. The formation of nitrotyrosine was evaluated by means of ELISA. Each value is presented as the mean ± SD from triplicate independent experiments. Significantly different from sham vehicle, # *p* < 0.05; UVA vehicle, * *p* < 0.05.

A whole-cell ELISA was applied to determine the effect of kinkéliba leaf extracts on UVR-induced nitrotyrosine. The concentrations of nitrotyrosine can be calculated from the standard curve created by 3NT-BSA (range: 10–2000 ng/mL). As shown in Figure 4, with the presence of UVA, nitrotyrosine was significantly increased compared with the sham vehicle (control cells). The nitrotyrosine levels in keratinocytes post-treated with 5 μg/mL kinkéliba leaf extract were significantly lower when compared with the UVA vehicle.

### 2.6. Kinkéliba Leaf Extract Formula Significantly Reduced Skin TEWL

**Figure 5:**
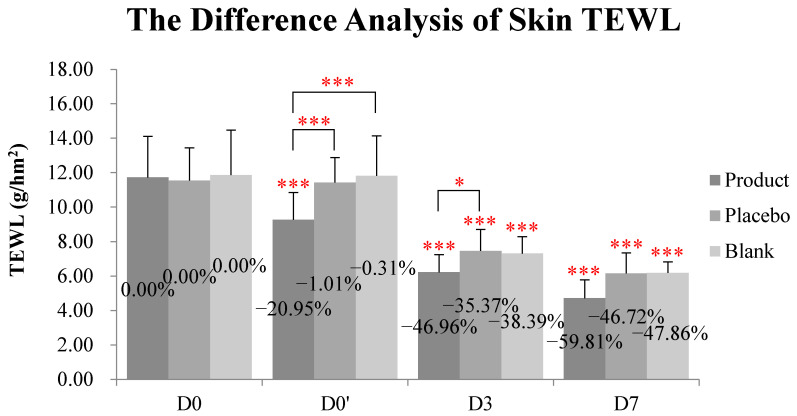
Reduction on skin TEWL by kinkéliba leaf extract was measured by VapoMeter, SWL5142. The lower the TEWL value was, the better the skin barrier repair ability would be. Significant differences between the baseline value and the measured values at each follow-up time in intra-group and inter-group: * *p* < 0.05, *** *p* < 0.001. D0: the day and time removing the patch; D0′: immediately after applying the test product; D3: three days after applying test product; D7: seven days after applying test product).

As shown in Table 1 and Figure 5, at D0′ (immediately after application), the decreases in the skin TEWL in the blank and placebo group were negligible (−0.31% and −1.01%, respectively). The kinkéliba leaf extract treatment (named as the test product group in table and figures) reduced the TEWL value by 20.95%, which is significant when compared with both the baseline and placebo group. At D3, the kinkéliba leaf extract treatment group’s skin TEWL was reduced by 49.96%, and it was also significantly better than the recovery of the placebo group. At D7, the kinkéliba leaf extract treatment group reduced the TEWL the most by 59.81%, but there were not significant differences between the kinkéliba leaf extract treatment, placebo, and blank groups.

### 2.7. Kinkéliba Leaf Extract Formula Reduced SLS-Irritated Erythema

**Figure 6:**
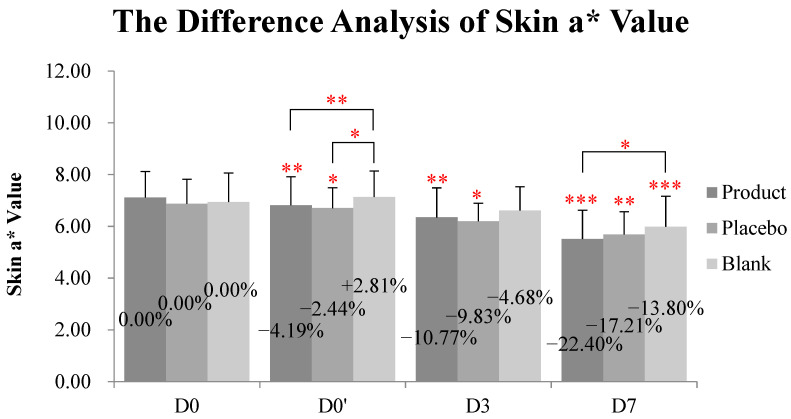
Reduction on skin a* value kinkéliba leaf extract was measured by Spectrophotometer, CM-2600d. The lower the value was, the lighter the skin redness after would be. Significantly differences between the baseline value and the measured values at each follow-up time in intra-group and inter-group: * *p* < 0.05, ** *p* < 0.01, *** *p* < 0.001. D0: the day and time removing the patch; D0′: immediately after applying the test product; D3: three days after applying test product; D7: seven days after applying test product.

At D0′ (immediately after application), both the kinkéliba leaf extract treatment (named as the test product group in table and figures) and the placebo group lowered the skin a* value compared with the baseline, and there were significant differences with the untreated group (Table 2 and Figure 6). At D3, the kinkéliba leaf extract treatment reduced skin a* value by 10.77%, compared with D0, but there were not significant differences with the placebo group. At D7, the skin a* value of all groups decreased significantly. The kinkéliba leaf extract treatment group presented better efficacies than other groups, but this difference is only significant when compared with the untreated blank group, not the placebo.

## 3. Discussion

Today, there is considerable interest in the application of ingredients from natural sources for the prevention of skin aging [36]. As a West African “long life” tea, the kinkéliba leaf’s health benefits on glucose-lowering or normalizing blood pressure have been validated both in vitro and in vivo [9,13]. It is worth mentioning that the increased levels of chronic pro-inflammatory cytokines are believed to drive many age-associated conditions, such as type 2 diabetes [15]. However, less is known about how such processes may contribute to skin aging. Our previous research on glucose-lowering capacity already presented kinkéliba’s involvement with anti-inflammation in other evaluation systems. Our results indicate that kinkéliba has great potential on treating skin inflammation and further investigations can examine how best to optimize the application and underlying mechanisms of action.

There are several types of pro-inflammatory cytokines related to skin inflammaging and photoaging. Interleukin-6 (IL-6) is a potent pro-inflammatory cytokine with a wide variety of biologic activities. It is produced by keratinocytes and the release is enhanced after UV light exposure [37]. It was reported that IL-6 acts as an important mediator of systemic sunburn reaction and plays an important role in the repair process [38]. Other research on dermal wounding suggested IL-6’s involvement in the modulation of keratinocyte differentiation, which is critical for epidermal stratification and the formation of a protective stratum corneum [39]. Keratinocyte differentiation dysfunction will increase trans-epidermal water loss (TEWL) and accelerate skin dryness, leading to a more pronounced appearance of fine lines and wrinkles. These studies indicate that the down-regulation of IL-6 plays an important role in skin healing and sunburn repair, as well as improvement of the skin barrier to retain skin hydration. Another key early response to ultraviolet B (UVB) by keratinocytes is the production of TNF-α [40,41]. This cytokine represents an important component of the inflammatory cascade in the skin and substantially promotes the activation of pro-MMP-2 in dermal fibroblasts embedded in type I collagen, leading to disruptive changes in the skin structure. Interleukin-8 (IL-8) is also considered as a major type of pro-inflammatory cytokine related to skin aging [42]. Recent work has revealed that IL-8 accumulation causes damage to the epidermal stem cells and affects the barrier function of the skin. IL-8′s level in the stratum corneum is also considered as a biomarker for monitoring the therapeutic effect in atopic dermatitis patients [43].

Taken together, reducing the levels of pro-inflammatory cytokines in stratum spinosum, stratum basale, and dermis is significant for the improvement of hydration and the water-retention capacity of stratum corneum [44,45]. In this study, UVA irradiation enhanced IL-6 and IL-8 levels in the human epidermal primary keratinocytes, while the kinkéliba leaf extract treatment significantly reduced UVA-induced increases in the IL-6 and IL-8 levels. However, there were no significant differences on the levels of TNF-α with or without kinkéliba extract treatment after UVA irradiation (data does not show). This might be due to the kinkéliba leaf extract targeting the modulation of keratinocytes differentiation more than apoptosis.

It is well known that inflammation is associated with the overproduction of ROS and RNS. Both ROS and RNS are key signaling molecules that play important roles in the progression of inflammatory disorders [46]. Increases in ROS/RNS levels trigger the production of pro-inflammatory cytokines in the skin cells, which will produce destructive changes which are seen as we age. According to previous publications, kinkéliba leaf extracts presented significant efficacies on free radical scavenging and the inhibition on inflammation. Its antioxidant properties were validated by different methods, including 1,1-diphenyl-2-picrylhydrazyl (DPPH), 2,2′-azinobis (3-ethylbenzothiazoline-6-sulphonic acid) (ABTS), hydroxyl radical, ferric thiocyanate, metal chelating activity, protein oxidation, and the β-carotene-linoleic bleaching assay [4]. Another study tested the antioxidant efficacy of several African plants and the kinkéliba leaf extract presented similar free radical scavenging levels with Vitamin E [1]. However, the data are all collected by cell-free assays, so further studies using cell-based systems are required to prove its cellular antioxidant activity.

ROS comprise a number of active metabolites, including hydroxyl radical, superoxide anion, and peroxyl radical, and their active precursors, namely singlet oxygen, hydrogen peroxide, and ozone [47]. In keratinocytes and fibroblasts, ROS were constantly generated and then removed rapidly by endogenous antioxidant substances. This process prevents harmful effects of ROS and maintains a pro-oxidant/antioxidant balance, resulting in cell stabilization [48]. However, the overproduction of ROS can be an issue. The application of extra antioxidants might be an effective strategy to reverse the deleterious effects of ROS generated by UVA [49]. In the present study, we demonstrated that UVA exposure caused a decrease in viability and an increase in ROS generation in human epidermal primary keratinocytes. The kinkéliba leaf extract pre-treatment protected UVA-induced damage through enhancing the decreased cell viability and anti-oxidative defense capability.

Reactive nitrogen species (RNS) are various nitric oxide-derived compounds, including nitroxyl anion, nitrosonium cation, higher oxides of nitrogen, S-nitrosothiols, and dinitrosyl iron complexes [50]. RNS also play a role in enhancing the genotoxic effects of UV radiation [51,52]. A previous study on HaCaT cells showed the increase in NO within minutes after UV radiation. However, NO is an unstable free radical species with a short half-life and is not that accurate to estimate [53,54]. Nitrotyrosine, a downstream of NO, is more likely to take longer time for the detection. Later detection of changes in nitrotyrosine provided an alternative way to evaluate earlier and more subtle increases in NO and peroxynitrite [55,56]. Another study also reported that nitrotyrosine only increased after other protective mechanisms have been overwhelmed. In this circumstance, the increases induced by UV radiation were evident at 4 h after irradiation and maximal at 16 h after UV radiation [57]. In this study, treatment with the kinkéliba leaf extract completely protected against a UVA-induced increase in nitrotyrosine. Therefore, the repair and protective and repair capacities of kinkéliba in the epidermis are linked with cellular defense systems, including the inhibition of reactive oxygen species (ROS) and reactive nitrogen species (RNS) in the UVA irradiation skin model.

To further validate kinkéliba’s anti-inflammatory benefit in vivo, a clinical trial was conducted and the efficacy of the kinkéliba leaf extract treatment was compared with the blank and placebo group. SLS was used to induce skin inflammation and barrier dysfunction [58]. In human volunteer participated studies, the topical application of kinkéliba has been demonstrated to alleviate SLS-induced redness, which indicates the repair of skin inflammation. Kinkéliba treatment also reduced skin TEWL more rapidly, compared with the blank and placebo groups. This data suggests that kinkéliba accelerated skin healing and provided a faster and better repair efficacy than the skin’s natural recovery process. More in vivo studies on kinkéliba’s protection of skin collagen would help to bring more understanding about its anti-inflammaging efficacy.

## 4. Materials and Methods

### 4.1. Vitexin and Isovitexin-Rich Kinkéliba Leaf Extract Preparation

The kinkéliba samples utilized for this study were gathered by the NGO, ASNAPP-Senegal, with members of the community, and with prior written consent from the community and community leaders. A certain amount of dry kinkéliba leaves was added into a beaker and 20 times the amount of purified boiling water was added. The leaves were soaked for 16 min before filtering. After repeating this 3 times, all extract aqueous solutions were collected for concentration. The raw extract was concentrated by rotary evaporation (60 °C) until its volume reached ¼ of its original volume, and then partitioned with petroleum ether, ethyl acetate, and *n*-BuOH, respectively, (equal volume for 3 times). After discarding all the petroleum ether, ethyl acetate, and *n*-BuOH layers, the water layer was evaporated in vacuo, freeze-dried, and named as “kinkéliba leaf extract” for subsequent experiments in this article.

HPLC determination was carried out on a Waters 2695 HPLC system with a DAD detector. The chromatographic separation of the analytes was achieved with a C18 column. The mobile phase consisted of water (A) and acetonitrile (B). The gradient program was as follows: 0–10 min with 25–46% acetonitrile (B); 11–30 min with 46–10% acetonitrile (B); 31–40 min with 10% acetonitrile (B). The flow rate of the mobile phase was maintained at 1 mL/min. The injection volume of sample solution was 10 μL.

### 4.2. Cell Culture, Kinkéliba Leaf Extract Treatment, and UVA Irradiation

Human primary epidermal keratinocytes were purchased from the American Type Culture Collection (Rockville, MD, USA). Keratinocyte at passages 1−5 were cultured in a dermal cell basal medium at 37 °C in 5% CO_2_. The culture medium was supplemented with the keratinocyte growth kit, which contained 0.4% bovine pituitary extract (BPE), 0.5 ng/mL rh TGF-α, 6 mM L-glutamine, 100 ng/mL hydrocortisone, 5 μg/mL insulin, 1.0 μM epinephrine, and 5 μg/mL apo-transferrin. 

The kinkéliba leaf extract was dissolved in a dimethyl sulfoxide (DMSO) solution at the concentration of 100 mg/mL as the stock solution. The stock solution was diluted to the desired final concentration using cell culture medium just before use. The final DMSO concentration did not exceed 0.1%. 

Semiconfluent cells (70%) were irradiated using a Longwave Ultraviolet Crosslinker (UVP CL-1000 L, Fisher Scientific, Carslbad, CA, USA), with a total of 4.5 J/cm^2^ UVA. UV energy setting is 900,000 mJ/cm^2^, repeating 5 exposures. All cells were treated identically throughout the procedure; the sham plate was shielded during irradiation. At the time of irradiation, the cells were washed with PBS, covered with a thin layer of PBS, and irradiated with UVA without a plastic lid. The intensity of UVA irradiation was measured using a UVA-365 radiometer (Lutron Co, Coopersburg, PA, USA).

### 4.3. Cell Viability Measurement

The cell survival rate was measured by the CCK-8 assay, according to the manufacturer’s instructions. For assays conducted using human primary epidermal keratinocytes, the cells were washed three times with PBS after the indicated treatment by kinkéliba leaf extract (5–50 μg/mL) or UVA irradiation. Subsequently, 10% of the CCK-8 solution in the culture medium was added to each well. After incubation for 2 h, absorbance was measured at 450 nm using VICtORX4 Multilabel Plate Reader (PerkinElmer, MA, USA). The values are expressed as the mean cell viability as a percentage of that of the vehicle DMSO(0.1% final volume)—treated cultures.

### 4.4. Measurement of Pro-Inflammatory Cytokines

The measurement of TNF-α, IL-6, and IL-8 expression was performed using reagents provided in the Human TNF-α ELISA Kit (ab181421), Human IL-6 ELISA Kit (ab178013), and Human IL-8 ELISA Kit (ab214030) (Abcam, Cambridge, MA, USA), respectively. Furthermore, 2 × 10^5^/well keratinocytes were seeded in 24-well plate with or without 50 μg/mL of kinkéliba leaf extract treatment 24 h before use, with adherent cells collected by scrape after UVA irradiation and the subsequent treatment by kinkéliba leaf extract. The subsequent measurements were conducted according to the manufacturer’s instruction.

### 4.5. Cellular Oxidative Stress (ROS) Measurement

The formation of ROS in cells was evaluated by means of DCFA corrected with the CCK-8 assay to account for cell loss after UVA radiation treatment, as described before. Briefly, keratinocytes were plated at a density of 15,000 cells per well in 96-well plates 24 h before use. The HEK cells were pre-treated for 24 h with 5–50 μg/mL kinkéliba leaf extract and then washed three times with PBS before UVA irradiation treatment. After irradiation by 4.32 J/cm^2^ UVA, fluorometric determination of intracellular ROS was estimated by loading the cells with 100 μL of DCFA (25 uM) in PBS for 30 min at 37 °C. The plates were placed in a VICTORX4 Multilabel Plate Reader (PerkinElmer, MA, USA). The fluorescence was monitored using an excitation wavelength of 485 nm and an emission wavelength of 535 nm. Individual absorbance values were corrected with cell viability with the CCK-8 assay before pooling. Each experimental point was performed in triplicate.

### 4.6. Measurement of Nitrotyrosine

The detection of nitrotyrosine was performed using reagents provided in a competitive ELISA kit ab113848 (Abcam, Cambridge, MA, USA). In addition, 3NT BSA was used as a standard positive control to validate the assays. In brief, 2 × 10^5^/well keratinocytes were seeded in a 24-well plate 24 h before use. The collection of adherent cells by scraping occurred at 16 h after UVA irradiation and subsequently treated with 5 μg/mL of the kinkéliba leaf extract. The cell pellet was solubilized in extraction buffer for 20 min on ice. After centrifuging at 4 °C for 20 min, the supernatants were collected into clean tubes. Each sample was diluted and adjusted to approximately the same protein concentration by a protein assay (Bio-Rad, Hercules, CA, USA). In total, 50 μL of each diluted standard or sample, together with 50 μL of 2× HRP detector antibody, was added to each well of nitrotyrosine-coated 96-well microplates and incubated for 2 h at room temperature. After washing four times, 100 μL of the HRP development solution was added to each well. The OD values were measured according to the manufacturer’s instructions. The concentrations of nitrotyrosine were calculated, taking into account the standard curve created by 3NT BSA.

### 4.7. In Vivo Study

A single-blind, randomized, self-comparison between the baseline value and the measured values was conducted after using kinkéliba leaf extract product, blank-controlled study, placebo-controlled study. In total, 0.1% kinkéliba leaf extract was formulated into gel for further clinical studies on ten subjects. Water was used as the placebo group. For the inner forearm, the test sites were divided into six test areas, A, B, C, D, E and F, each of which was about 4 cm × 5 cm in area. The dermatologist observed and selected the three most suitable erythema areas for measurement. According to the randomization sheet, the test product application area, placebo control area, and blank control area were defined.

On D-2*, the dermatologist observed the skin condition of both inner forearms. According to the inclusion and exclusion criteria, the dermatologist screened the subjects who met the requirements. After the subjects were successfully enrolled, the technician divided the subjects’ skin (both inner forearms) into six test areas, then applied standard patch testing materials with 3% SLS solution (liquid, drip onto the filter paper attached to standard patch testing materials) on six test areas and stuck to the subjects’ forearms by medical adhesive tape for 24 h. On D-1*, the technician removed the patch applications and urged the subjects not to do anything that would affect the test results, such as grabbing the test area. On D0, D0′*, D3*, and D7*, the skin TEWLs were measured with VapoMeter (SWL5142), and chromameter (Spectrophotometer CM-2600d) was used to measure a* value for SLS-induced redness, in terms of the erythema index.

(*D-2: 2 days before measuring; D-1: 24 h after patch application; D0: the day after removing the patch; D0′: immediately after applying the test product; D3: 3 days after applying test product; D7: 7 days after applying test product.)

### 4.8. Statistical Analysis

For the in vitro study, data were presented as normalized results from a minimum of three independent experiments. Significant differences in the cell survival rate, ROS, and nitrotyrosine were determined by one-way ANOVA followed by the Tukey’s multiple comparisons test using the Graphpad Prism 5 software package (GraphPad Software, San Diego, CA, USA).

For the clinical study, an analysis of the differences between the baseline value (D0) and the measured values at each follow-up time (D0′/D3/D7) intra-group were performed by repeated-measurement ANOVA tests. Furthermore, the inter-group difference analysis between the test product group and the placebo control group/blank control group at each follow-up time were performed by multivariate analysis of variance tests. SPSS 21.0 was used for the data analysis. *p*-values < 0.05 were considered significant. The rate of each parameter’s change is calculated as follows:

(1) $$\mathrm{Rate}\;\mathrm{of}\;\mathrm{change}\;\left(\%\right)=\frac{\mathrm{The}\;\mathrm{measured}\;\mathrm{value}\;\mathrm{at}\;\mathrm{follow}-\mathrm{up}\;\mathrm{time}\;-\;\mathrm{Baseline}\;\mathrm{value}}{\mathrm{Baseline}\;\mathrm{value}\;}\times 100\%$$

## 5. Conclusions

In summary, the effect of kinkéliba leaf extracts was investigated in normal human primary keratinocytes and in vivo. After 24 h treatment, the kinkéliba leaf extract significantly enhanced the cell survival rate reduced by UV irradiation. Its elimination on UV-induced cellular ROS/RNS was demonstrated for the first time in human primary skin cells, while previous research only presented kinkéliba’s free radical scavenging efficacy in the cell-free system. Although our previous research on glucose-lowering capacity already demonstrated kinkéliba’s involvement with anti-inflammation in other evaluation systems, this study validated kinkéliba’s capacity to reduce UV exposure generated pro-inflammatory cytokines IL-6/8 and alleviate skin inflammation. As a traditional herbal tea with plenty of health benefits, kinkéliba has great potential in the field of personal care. This research provided a new application direction for kinkéliba leaf extracts as an active ingredient in skincare and expanded its traditional applications in beverage and nutraceuticals.

## 6. Patents

Hu, S.; Simon, J.E.; Wu, Y.; WANG, M.; Wu, Q. Use of *Combretum micranthum* extract in cosmetics: U.S. Patent Application 17/275,087[P] resulted in part from the research reported in this manuscript.

## Figures and Tables

**Table 1:** Difference analysis of skin TEWL.

Skin TEWL (g/hm^2^)	Test Product Group	Placebo Control Group	Blank Control Group	*p* (Inter-Group)		
				Product vs. Placebo	Product vs. Blank	Placebo vs. Blank
D0 baseline	11.73 ± 2.39	11.54 ± 1.90	11.86 ± 2.61			
D0′	9.27 ± 1.59	11.43 ± 1.46	11.82 ± 2.32			
D3	6.22 ± 1.03	7.46 ± 1.25	7.31 ± 0.98			
D7	4.71 ± 1.07	6.15 ± 1.20	6.18 ± 0.64			
*p* (D0′ vs. D0)	<0.001	0.697	0.832	<0.001	<0.001	0.814
*p* (D3 vs. D0)	<0.001	<0.001	<0.001	0.042	0.294	0.550
*p* (D7 vs. D0)	<0.001	<0.001	<0.001	0.099	0.286	0.782

The skin TEWL was measured by VapoMeter, SWL5142. The lower the TEWL value was, the better the skin barrier repair ability would be. The results of the skin TEWL were presented as mean ± SD, and the number of subjects was 10. The intra-group difference analysis between the baseline values and the measured values at each follow-up time were performed with a repeated-measurement ANOVA test. The inter-group difference analyses between the three groups at each follow-up time were performed with a multivariate analysis of variance test; D0: the day removing the patch; D0′: immediately after applying the test product; D3: three days after applying the test product; D7: seven days after applying the test product. *p* values < 0.05 (with significant difference) were marked as red.

**Table 2:** Difference analysis of skin a* value.

Skin a* Value	Test Product Group	Placebo Control Group	Blank Control Group	*p* (Inter-Group)		
				Product vs. Placebo	Product vs. Blank	Placebo vs. Blank
D0 baseline	7.11 ± 1.01	6.87 ± 0.95	6.94 ± 1.12			
D0′	6.81 ± 1.10	6.70 ± 0.79	7.13 ± 1.00			
D3	6.35 ± 1.14	6.19 ± 0.70	6.61 ± 0.92			
D7	5.52 ± 1.11	5.69 ± 0.88	5.98 ± 1.18			
*p* (D0′ vs. D0)	0.006	0.039	0.129	0.243	0.003	0.016
*p* (D3 vs. D0)	0.004	0.016	0.167	0.770	0.149	0.280
*p* (D7 vs. D0)	<0.001	0.004	<0.001	0.268	0.028	0.549

Skin colors as L*, a*, and b* were measured by Spectrophotometer, CM-2600d. The a* was used to evaluate the skin redness. The lower the value was, the lighter the skin redness after would be. The a* values were presented as mean ± SD, and the number of the subjects was 10. The intra-group difference analysis between the baseline value and the measured values at each follow-up time were performed with a repeated-measurement ANOVA test. The inter-group difference analysis between the three groups at each follow-up time were performed with a multivariate analysis of variance test. *p* values < 0.05 (with significant differences) were marked as red.

## Data Availability

Data available on request due to restrictions (privacy). The data presented in this study are available on request from the corresponding author. The data are not publicly available due to privacy.

## Supplementary Materials

The following supporting information can be downloaded at: https://www.mdpi.com/article/10.3390/molecules28041791/s1, Figure S1: Human primary keratinocytes were treated with different dosage of UV radiation. Cell viability was determined by the CCK-8 assay. Each value is presented as the mean ± SD from triplicate independent experiments. Significantly different from sham vehicle; UVA vehicle, *** *p* < 0.001; Figure S2. The chromatograms of A: Vitexin standard; B: Isovitexin standard; C: Kinkéliba leaf extract.

## Sample Availability

Samples of the compounds are not available from the authors.
