# Supplementary material for: Kinkéliba (Combretum micranthum) Leaf Extract Alleviates Skin Inflammation: In Vitro and In Vivo Study
Source: Molecules. 2023 Feb 14;28(4):1791. doi: 10.3390/molecules28041791 (PMC9964726; doi:10.3390/molecules28041791)
Supplement: Supplementary file 1 [file molecules-28-01791-s001.zip › molecules-2151371-supplementary.pdf]

Supplementary Materials

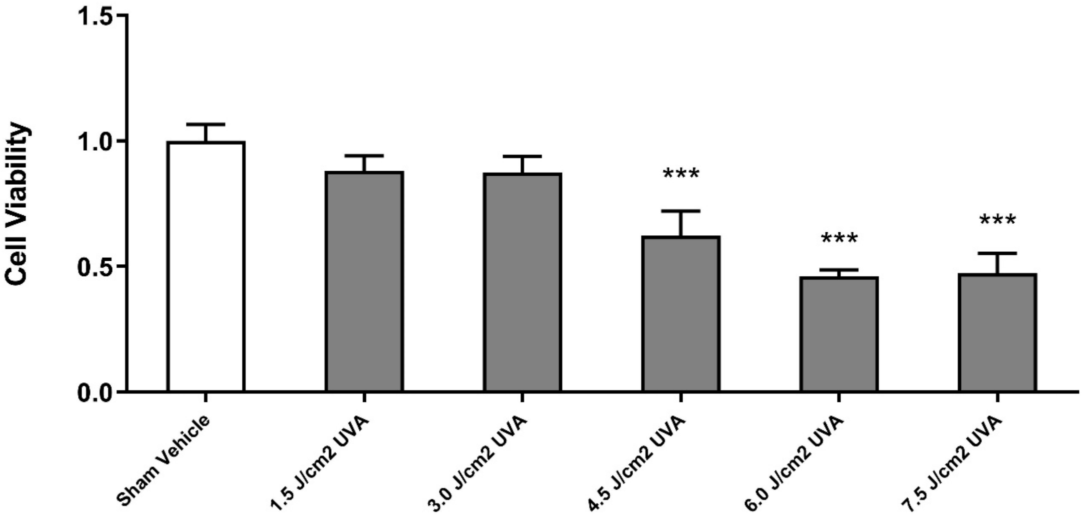

Figure S1. Human primary keratinocytes were treated with different dosage of UV radiation. Cell viability was determined by the CCK-8 assay. Each value is presented as the mean ± SD from triplicate independent experiments. Significantly different from sham vehicle; UVA vehicle, *** p <0.001.

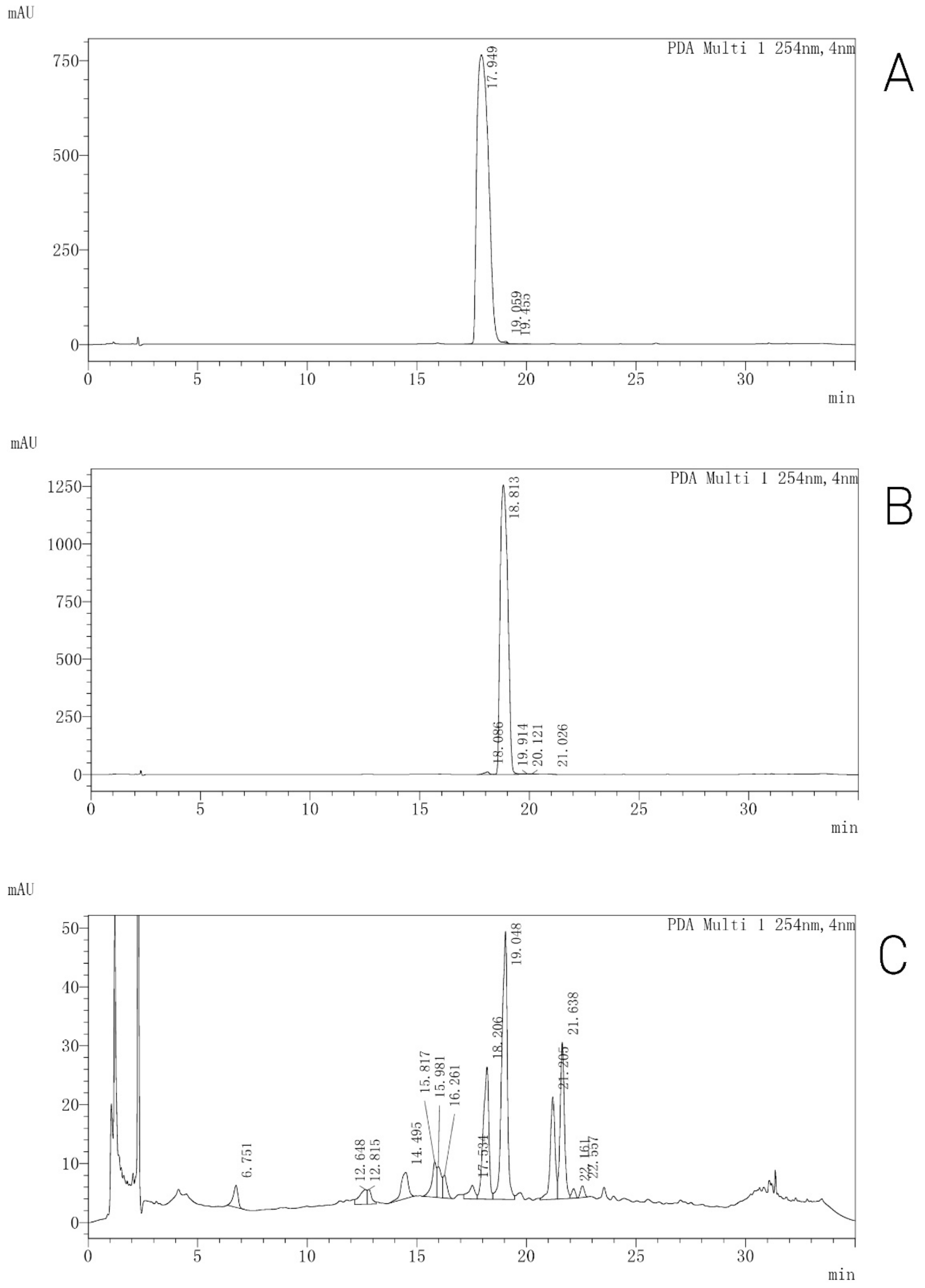

Figure S2. The chromatograms of **A**: Vitexin standard; **B**: Isovitexin standard; **C:** Kinkéliba leaf extract.
